# Cardiac extracellular volume quantified with T1 mapping techniques reflects degree of cardiac and neurological involvement in Hereditary Transthyretin Amyloidosis

**DOI:** 10.1186/1750-1172-10-S1-P44

**Published:** 2015-11-02

**Authors:** Esther González-López, María Gallego-Delgado, Francisco Muñoz-Beamud, Juan Buades, Lucía Galán, Jose Luis Muñoz Blanco, Jesús González-Mirelis, Pablo García-Pavía

**Affiliations:** 1 Hospital Puerta de Hierro, Cardiology, 28222, Majadahonda, Spain; 2 Hospital Juan Ramón Jiménez, Internal Medicine, 21005, Huelva, Spain; 3 Hospital Sont Llàzer, Internal Medicine, 07198, Palma de Mallorca, Spain; 4 Hospital Clínico San Carlos, Neurology, 28040, Madrid, Spain; 5 Hospital Gregorio Marañón, Neurology, 28007, Madrid, Spain

## Background

Amyloidotic cardiomyopathy (AC) in Hereditary Transthyretin Amyloidosis (ATTR) determines prognosis and treatment options. Cardiac Magnetic Resonance (CMR) has shown its utility in the diagnosis and characterization of AC. Moreover, CMR T1 mapping techniques are useful to assess myocardial extracellular volume (ECV) fraction in AC.

We hypothesized that ECV allows identification of AC in ATTR patients and that there is a correlation between cardiac ECV and the degree of neurological impairment caused by TTR amyloid extracardiac deposits.

## Methods

31 genetic proven ATTR patients at different stages of the disease (19 males; mean age 49±12 years; 26 with Val30Met mutation) underwent a T1 mapping CMR study and a neurological evaluation with NIS-LL score (sensitive, motor and reflex examination), Norfolk-QOL questionnaire (symptoms and quality of life) and Karnofsky index (general health status). AC was defined by positive 99mTc-DPD scintigraphy (uptake grade>2) or left ventricular hypertrophy >12mm. with typical gadolinium kinetics/enhancement of amyloidosis at CMR in the absence of DPD-scan (9 patients).

## Results

5 patients had AC (all of them determined by scintigraphy). Mean ECV was increased in patients with AC (0.490±0.131 vs. 0.289±0.035; P=0.026). ECV correlated with age (R=0.467; P=0.008), NTproBNP (RS=0.846; P<0.001), maximum wall thickness (R=0.621; P<0.001), left ventricular mass index (R=0.685; P<0.001), left ventricular ejection fraction (R=-0.378;P=0.036), NIS-LL (RS=0.604; P=0.001), Norfolk-QOL (RS=0.529; P=0.003) and Karnofsky (RS=-0.517; P=0.004). A cut-off value of ECV=0.357 calculated by ROC curve, was diagnostic of AC with 100% sensibility and specificity (P<0.001). ECV and NTproBNP values were the only cardiac parameters that significantly correlated with neurological scores.

## Conclusions

ECV quantification by CMR allows identification of AC in ATTR and correlates with the degree of neurological impairment. This non-invasive technique could be a useful tool for early diagnosis and to track cardiac and extracardiac amyloid disease.

